# Up-Regulation of Mitochondrial Antioxidant Superoxide Dismutase Underpins Persistent Cardiac Nutritional-Preconditioning by Long Chain *n*-3 Polyunsaturated Fatty Acids in the Rat

**DOI:** 10.3390/jcm5030032

**Published:** 2016-03-04

**Authors:** Grace G. Abdukeyum, Alice J. Owen, Theresa A. Larkin, Peter L. McLennan

**Affiliations:** 1 Division of Medical and Exercise Science, School of Medicine, Faculty of Science Medicine and Health, University of Wollongong, Wollongong NSW 2522, Australia; Grace.Abdukeyum@hnehealth.nsw.gov.au; 2 Centre of Cardiovascular Research & Education in Therapeutics, School of Public Health & Preventive Medicine, Monash University, Melbourne VIC 3004, Australia; alice.owen@monash.edu; 3 Centre for Human and Applied Physiology, Graduate School of Medicine, School of Medicine, Faculty of Science Medicine and Health, University of Wollongong, Wollongong NSW 2522, Australia; tlarkin@uow.edu.au

**Keywords:** fish oil, preconditioning, antioxidant, reactive oxygen species, ischaemia, reperfusion, *n*-3 PUFA, lipid oxidation, infarct

## Abstract

Reactive oxygen species paradoxically underpin both ischaemia/reperfusion (I/R) damage and ischaemic preconditioning (IPC) cardioprotection. Long-chain omega-3 polyunsaturated fatty acids (LC*n*-3 PUFA) are highly susceptible to peroxidation, but are paradoxically cardioprotective. This study tested the hypothesis that LC*n*-3 PUFA cardioprotection is underpinned by peroxidation, upregulating antioxidant activity to reduce I/R-induced lipid oxidation, and the mechanisms of this nutritional preconditioning contrast to mechanisms of IPC. Rats were fed: fish oil (LC*n*-3 PUFA); sunflower seed oil (*n*-6 PUFA); or beef tallow (saturated fat, SF) enriched diets for six weeks. Isolated hearts were subject to: 180 min normoxic perfusion; a 30 min coronary occlusion ischaemia protocol then 120 min normoxic reperfusion; or a 3 × 5 min global IPC protocol, 30 min ischaemia, then reperfusion. Dietary LC*n*-3 PUFA raised basal: membrane docosahexaenoic acid (22:6*n*-3 DHA); fatty acid peroxidisability index; concentrations of lipid oxidation products; and superoxide dismutase (MnSOD) activity (but not CuZnSOD or glutathione peroxidase). Infarct size correlated inversely with basal MnSOD activity (*r*^2^ = 0.85) in the ischaemia protocol and positively with I/R-induced lipid oxidation (lipid hydroperoxides (LPO), *r*^2^ = 0.475; malondialdehyde (MDA), *r*^2^ = 0.583) across ischaemia and IPC protocols. While both dietary fish oil and IPC infarct-reduction were associated with reduced I/R-induced lipid oxidation, fish oil produced nutritional preconditioning by prior LC*n*-3 PUFA incorporation and increased peroxidisability leading to up-regulated mitochondrial SOD antioxidant activity.

## 1. Introduction

Regular consumption of fish or fish oil reduces cardiovascular mortality [1], often without modifying classical risk factors. For example, sudden death is reduced in high-risk post-MI patients, without significant reductions in blood pressure, blood lipids or prevention of new cardiac events [2]. This cardioprotection, associated with omega-3 long-chain polyunsaturated fatty acid (LC*n*-3 PUFA) consumption, is observed independently of the prevention of ischaemic events [3], and therefore supports a cardiac origin related to incorporation of the fatty acids into myocardial membranes [4]. Ischaemic preconditioning (IPC) is a powerful cardioprotective process, wherein brief periods of ischaemia, insufficient to produce cellular damage, can protect the myocardium from the damaging effects of a subsequent more prolonged ischaemic insult. The protective envelope of IPC is twofold, categorised as: classical or early preconditioning, which provides cardioprotection for several hours after the IPC stimulus; with a second phase called late or delayed preconditioning which occurs 24–72 h after the stimulus. Experimentally, the LC*n*-3 PUFA confer their cardioprotection in part through nutritional preconditioning of the myocardium that in rat heart is at least as effective in reducing infarct size [5,6] and promoting post-ischaemic contractile recovery [5], and more effective in preventing ischaemia or reperfusion induced cardiac arrhythmias [5], as early ischaemic preconditioning (IPC). Classical, early IPC cardioprotection disappears within several hours of the initial preconditioning stimulus and repeated brief preconditioning episodes become ineffective in providing this early protection [7]. In contrast, the cardioprotection derived from dietary LC*n*-3 PUFA is obtained only after they are incorporated into and continuously present in the myocardium for at least seven days and it persists over weeks or months, for however long elevated membrane content is sustained [5,8,9]. There is no desensitisation apparent, with acute ischaemia or reperfusion arrhythmias prevented after five weeks [8] to 52 weeks [10] of continuous exposure to dietary LC*n*-3 PUFA. Therefore, the cardioprotective benefit of fish oil appears to mimic the more sustained, repeatable protection of late IPC and other persistent preconditioning stimuli [11,12,13]. Moreover, the LC*n*-3 PUFA [5] share with late IPC [14] the capacity to protect against both infarction and myocardial stunning.

Myocardial ischaemia and reperfusion (I/R) stimulates production of reactive oxygen species (ROS) and depletes antioxidants in the heart, creating oxidative stress, oxidation of biomolecules and cell damage. Paradoxically, these free radicals also act as triggers of IPC [15,16]. The highly unsaturated LC*n*-3 PUFA found in fish oil: 20:5*n*-3 eicosapentaenoic acid (EPA) and 22:6*n*-3 docosahexaenoic acid (DHA) with their numerous bisallylic carbon atoms, are more susceptible to peroxidation and generation of damaging reactive oxygen species than are shorter, less unsaturated fatty acids such as 18:2*n*-6 linoleic acid and 20:4*n*-6 arachidonic acid [17], which raises the prospect of adverse effects of fish oil supplementation. However, there is no clinical evidence to suggest that fish oil supplementation or high fish diets promote oxidative stress-related cardiovascular disease. In contrast, production of ROS is a mechanism invoked to explain the paradoxical effects of late IPC in the heart, which works by inducing upregulation of endogenous antioxidant protective mechanisms [16,18]. That protection occurs in lieu of the extreme oxidation of biomolecules and cell damage that usually occurs with I/R-induced oxidative stress and antioxidant depletion.

The current study tested the hypothesis that incorporation of LC*n*-3 PUFA into myocardial membranes increases their peroxidation potential and basal fatty acid oxidation, which by their constant presence, in turn increases endogenous antioxidant enzymes to confer physiological cardioprotective actions against I/R-stimulated oxidative stress. We propose that this will contrast to the mechanism of early IPC cardioprotection.

## 2. Experimental Section

### 2.1. Animals and Diets

Fifty-four male Wistar rats were randomly assigned to three experimental dietary groups. For six weeks they were fed one of three iso-energetic diets containing either predominantly saturated animal fat, *n*-6 PUFA or LC*n*-3 PUFA as the source of fat. The diets were based on the American Institute of Nutrition AIN93 rat diet, containing all essential vitamins and minerals with gelatine as a component of the protein source. The diet was prepared with 10% (dry wt) fat (23% metabolisable energy as fat) consisting of: 7% beef tallow plus 3% olive oil (SF diet); 5% sunflower seed oil plus 5% olive oil (*n*-6 PUFA diet); or 7% fish oil (Nu-Mega high DHA tuna fish oil) plus 3% olive oil (LC*n*-3 PUFA diet). In addition to delivering diets rich in saturated fat, *n*-*6* PUFA or *n*-3 PUFA, the oil blends in the LC*n*-3 PUFA diet and the *n*-6 PUFA diet were designed to deliver similar total PUFA, and the oil blends in the LC*n*-3 PUFA diet and the SF diet were designed to deliver similar total *n*-6 PUFA, as previously described [5]. All diets contained sufficient PUFA to prevent essential fatty acid deficiency [5].

Animal care and experiments were conducted with the approval of the University of Wollongong, Animal Care and Ethics Committee according to the guidelines of the National Health and Medical Research Council, Australia, Australian Code of Practice for the Care and Use of Animals for Scientific Purposes [19].

### 2.2. Heart Preparation

After six weeks of feeding, rats were anaesthetised (pentobarbital sodium, 60 mg·kg^−1^ i.p.), the thorax was opened, the heart was rapidly excised, submerged in ice-cold perfusate to arrest beating, and immediately perfused by an aortic cannula in the Langendorff mode at a constant pressure of 75 mmHg delivering warm (37 °C) Krebs–Henseleit bicarbonate buffer gassed with 5% CO_2_ in O_2_ [5]. The left atrium was opened and a thin-walled balloon catheter was introduced into the left ventricle, with balloon volume adjusted to maintain end diastolic pressure of 6–8 mmHg. A 6–0 silk suture was passed through the myocardium closely underlying the left anterior descending coronary artery near its origin.

### 2.3. Index Ischaemia and Ischaemic Preconditioning

**Figure 1:**
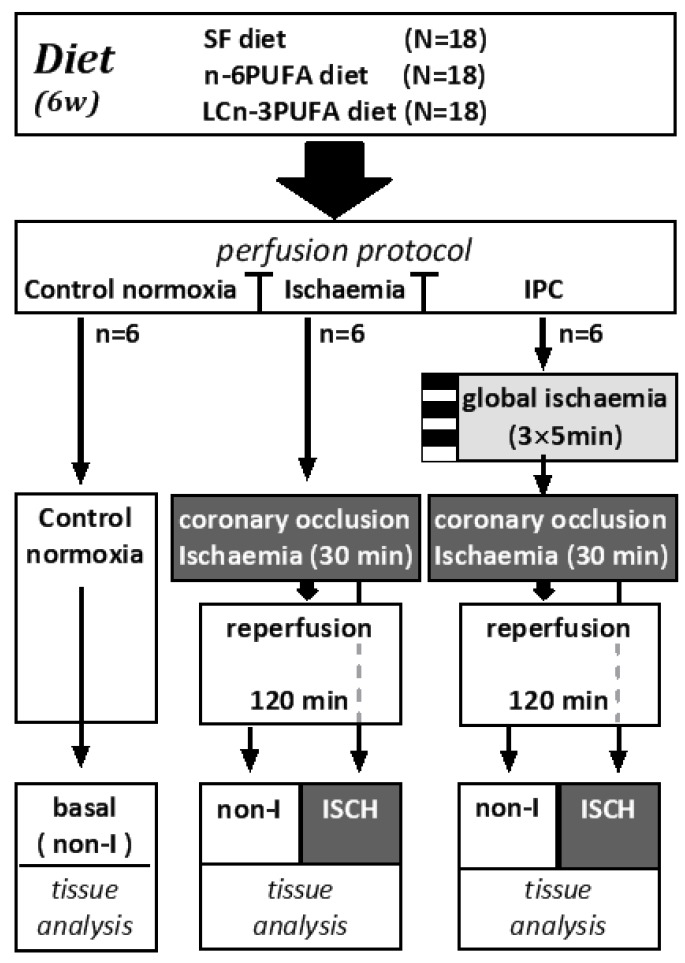
Flow chart illustrating the distribution of dietary groups into: Control normoxic; Ischaemia; and IPC perfusion protocols. In each protocol, isolated hearts were perfused for 180 min. Ischaemia protocol and IPC protocol hearts were dissected into non-ischaemic (non-I) and ischaemic (ISCH) tissue for biochemical analysis. IPC: ischaemic preconditioning.

Each dietary group (*n* = 18) was separated into groups of *n* = 6 and randomly assigned to one of three perfusion protocols for 180 min after initial 30 min equilibration perfusion (Figure 1).

1. Control normoxia protocol (*n* = 6 per diet): Hearts were perfused throughout with oxygenated Krebs–Henseleit solution.
2. Ischaemia protocol (n = 6 per diet): Hearts were normoxically perfused for 30 min followed by 30 min index-ischaemia and 120 min normoxic reperfusion. Index-ischaemia was induced by occluding the left anterior descending coronary artery.
3. Ischaemic preconditioning (IPC) protocol (n = 6 per diet): Hearts were subjected to three cycles of five minutes global ischaemia (zero perfusion), each followed by five minutes normoxic reperfusion, prior to the 30 min index-ischaemia then 120 min normoxic reperfusion [5].

On completion of 120 min reperfusion in the ischaemia and IPC protocols, the coronary artery was re-occluded to reveal the ischaemic zone at risk (I-z/r). Hearts were then cut into 2 mm slices. The central slice was incubated in a buffer containing triphenyl-tetrazolium chloride and sodium phosphate (pH 7.4), then stored in 10% formalin until photographed and analysed for infarct size. Infarct size was reported as a percentage of the zone at risk. The remaining slices were separated into non-ischaemic (non-I) and ischaemic (ISCH) segments (Figure 1). Samples of fresh ISCH and non-I tissue were used immediately for lipid hydroperoxide (LPO) analysis, with the remainder rapidly frozen and stored at −80 °C for analysis of other markers of oxidation and antioxidant status. Samples of control normoxic heart were always taken from the left ventricle anterior free wall, supplied by the left anterior descending coronary artery, that would have been subject to ischaemia in the other protocols. It represents the basal state of the ischaemic zone at risk.

### 2.4. Measurement of Oxidative Stress Biomarkers

Concentrations of LPO were measured by modification of the ferric thiocyanate assay using a colorimetric assay kit (Lipid Hydroperoxide Assay, Cayman Chemical Company, Ann Arbor, MI, USA) and were expressed per mg of protein. Concentrations of malondialdehyde (MDA) were measured in thawed tissue homogenates by reverse-phase HPLC with fluorescence detection [20].

### 2.5. Measurement of Antioxidants

*Endogenous:* Total superoxide dismutase (SOD) activity and CuZnSOD activity were measured in ventricle sections of: ISCH tissue; and non-I tissue using a BIOXYTECH^®^-SOD-525™ assay kit (Oxis Research™, Portland, OR, USA). The activity of mitochondrial SOD activated by manganese (MnSOD) was calculated as the difference between total SOD and CuZnSOD. The activity of SOD was expressed per mg of tissue protein. Glutathione peroxidase (GPX) activity was measured in ventricle sections of: ISCH; and non-I ventricle using BIOXYTECH^®^GPx-340TM assay kit (OxisResearch™, Portland OR, USA) and was expressed per mg of tissue protein. *Exogenous:* Myocardial vitamin E (alpha-tocopherol) was measured by HPLC with electrochemical detection, using a modification of the method described by Yang [21].

### 2.6. Myocardial Fatty Acid Analyses

Total lipids were extracted from 100 to 200 mg samples of ventricular myocardium using a modification of the Folch method [22]. Phospholipids were isolated from the total muscle lipid by solid phase extraction using silica Sep-pak™ cartridges (Waters, Rydalmere, NSW, Australia). Fatty acid methyl esters were prepared by direct transesterification of the phospholipid fraction [23] and analysed by gas chromatography using a Shimadzu GC-17A with flame ionization detection using a 30 m × 0.25 mm, 0.25 μm FAMEWAX column (J and W Scientific, Santa Clara CA, USA) with hydrogen as carrier gas and a step temperature program rising from 150 °C to 260 °C, over 27 min and held for 6 min. Individual fatty acids were identified by their retention times with reference to authentic fatty acid methyl ester standards (Sigma-Aldrich, Rydalmere, NSW, Australia) and expressed as a percentage of total phospholipid fatty acids.

### 2.7. Statistical Analyses

Results were expressed as mean ± SEM. Data were analysed by two-way analysis of variance (ANOVA) for diet and treatment main effects (normoxic perfusion, ischaemia, IPC + ischaemia) and by multi-way ANOVA for diet, treatment and ISCH *versus* non-I tissue main effects. Tukey’s HSD test was used for *post-hoc* pairwise comparison of individual means and interactions. Within dietary groups, ISCH and non-I sections of the same hearts were compared using repeated measures ANOVA. Statistical analyses were performed using Statistix software, version 10 (Analytical Software, Tallahassee, FL, USA). Linear regression analysis with Pearson’s correlation was performed to determine linear associations between lipid oxidation products, antioxidants and infarct size using Prism for Windows, version 6 (GraphPad Software, La Jolla, CA, USA). Statistical significance was accepted at *p* < 0.05.

## 3. Results

Neither the starting body weight, the final body weight nor the change in body weight over six weeks differed between dietary groups (Start: SF 348 ± 6 g; *n*-6 PUFA 351 ± 5 g; LC*n*-3 PUFA 352 ± 6 g. Six weeks: SF 460 ± 11 g; *n*-6 PUFA 457 ± 8 g; LC*n*-3 PUFA 480 ± 9 g. Change: SF 112 ± 7 g; *n*-6 PUFA 109 ± 9 g; LC*n*-3 PUFA 128 ± 7 g. *n* = 18 per diet).

### 3.1. Myocardial Membrane Phospholipid Fatty Acid Composition

The relative concentration of DHA (22:6*n*-3) was greater in phospholipid of LC*n*-3 PUFA hearts than in either *n*-6 PUFA or SF hearts (*p* < 0.05) (Table 1). The LC*n*-3 PUFA hearts contained significantly lower concentrations of linoleic (18:2*n*-6) and arachidonic acids (20:4*n*-6) compared with *n*-6 PUFA or SF hearts. Total *n*-3 PUFA was greater in LC*n*-3 PUFA hearts compared with either *n*-6 PUFA or SF hearts, and lower in *n*-6 PUFA than SF hearts (*p* < 0.05). The total concentration of *n*-6 PUFA was lower in LC*n*-3 PUFA hearts than in either *n*-6 PUFA or SF hearts and greater in *n*-6 PUFA than SF hearts (*p* < 0.05).

**Figure 2:**
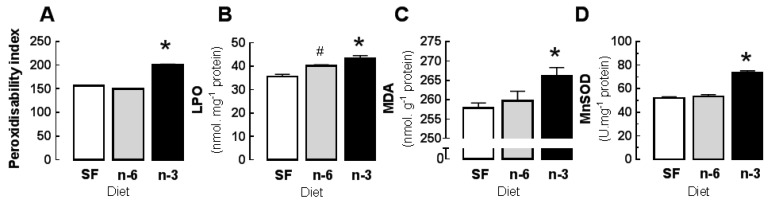
Influence of six weeks dietary fat feeding on basal: (**A**) membrane fatty acid peroxidisability index; and concentrations of (**B**) lipid hydroperoxides (LPO); (**C**) malondialdehyde (MDA); and (**D**) antioxidant superoxide dismutase (MnSOD) of basal or non-I regions of the heart after 180 min of isolated perfusion protocol. Open columns (SF): saturated fat diet; shaded columns (*n*-6): *n*-6 PUFA rich diet; filled columns (*n*-3): LC*n*-3 PUFA rich fish oil diet. Values are means ± SEM. *n* = 18 per dietary group except peroxidisability index: *n* = 6. * different from both other diet groups, *p* < 0.05. ^#^ different from SF group, *p* < 0.05.

No significant dietary differences were observed in the membrane phospholipid total saturated fatty acids or total PUFA. The SF hearts had greater concentrations of total monounsaturated fatty acids (MUFA). Membrane unsaturation index (UI) and peroxidisability index (Figure 2A) were significantly greater in LC*n*-3 PUFA than in either *n*-6 PUFA or SF hearts (*p* < 0.05), which were not significantly different from each other.

### 3.2. Basal Properties: Effects of Diet on Oxidative Stress and Antioxidant Activity

The basal and non-I tissue derived from the three perfusion protocols exhibited no significant between protocol differences in tissue concentrations of lipid oxidation products LPO or MDA or anti-oxidants within any dietary group (pooled data shown in Figure 2). This establishes the non-I measures as representative of the basal state of the ISCH region.

**Figure 3:**
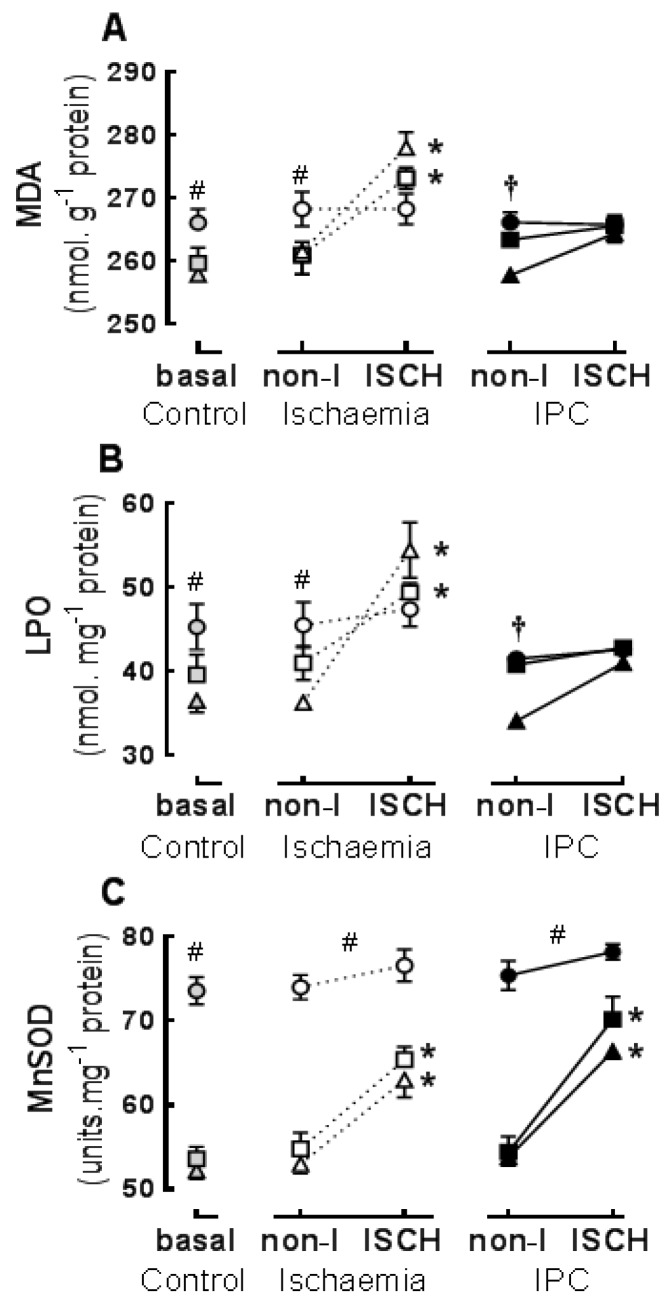
Influence of six weeks dietary fat feeding on cardiac lipid oxidation and antioxidant markers in ischaemic (ISCH) or non-ischaemic (non-I) regions after: Control normoxic perfusion (basal), Ischaemia perfusion, or ischaemic preconditioning (IPC) perfusion protocols: (**A**) lipid hydroperoxides (LPO); (**B**) malondialdehyde (MDA); and (**C**) superoxide dismutase (MnSOD). Data are from hearts that were normoxic throughout (basal), or the non-I and ISCH regions of hearts subjected to 30 min of regional ischaemia with or without prior IPC. Diet groups: ▲▲Δ—saturated fat (SF); ■■□—*n*-6 PUFA; ●●○—LC*n*-3 PUFA. Values are means ± SEM. *n* = 18 per diet, *n* = 6 per perfusion protocol. * ISCH different from non-I region within diet, *p* < 0.05; ^#^ *n*-3 PUFA different from SF *p* < 0.05; † LC*n*-3 PUFA and *n*-6 PUFA different from SF.

The concentrations of LPO in basal and non-I tissue were significantly greater in LC*n*-3 PUFA than in either *n*-6 PUFA or SF hearts and greater in *n*-6 PUFA than SF hearts (*p* < 0.05) (Figure 3A). The concentrations of MDA in basal and non-I tissue were significantly greater in LC*n*-3 PUFA than in either *n*-6 PUFA or SF hearts (*p* < 0.05), which were not different from each other (Figure 3B).

The activity of MnSOD in basal and non-I tissue was significantly greater in LC*n*-3 PUFA hearts, than in SF or *n*-6 PUFA hearts (Figure 3C). In basal and non-I tissue there were no significant dietary differences in CuZnSOD activity (basal, non-I (U·mg^−1^ protein): SF 15.3 ± 0.9; *n*-6 PUFA 16.9 ± 0.7; LC*n*-3 PUFA 17.4 ± 0.6 *n* = 18) (*p* > 0.05) or GPX (basal, non-I (mU·mg^−1^ protein): SF 19.2 ± 1.5; *n*-6 PUFA 19.7 ± 1.5; LC*n*-3 PUFA 21 ± 1.2 *n* = 15). The concentration of α-tocopherol was significantly greater in *n*-6 PUFA hearts than in either LC*n*-3 PUFA or SF hearts (*p* < 0.05) (basal, non-I (μM): SF 6.1 ± 0.4; *n*-6 PUFA 6.9 ± 0.2; LC*n*-3 PUFA 5.9 ± 0.6 *n* = 15).

### 3.3. Ischaemic Responses: Effects of Diet and Ischaemic Preconditioning on Oxidative Stress and Antioxidant Capacity in Hearts Subjected to Regional I/R

*Ischaemia*: The concentrations of LPO (Figure 3A) and MDA (Figure 3B) were acutely increased in the ISCH compared to non-I region of *n*-6 PUFA and SF hearts (*p* < 0.01) but not in LC*n*-3 PUFA hearts. The concentrations of LPO and MDA in the ISCH region were significantly greater in SF hearts than in LC*n*-3 PUFA hearts (Figure 3A,B).

*IPC*: There were no significant acute changes in LPO or MDA in ISCH compared to non-I regions within any dietary group (Figure 3A,B), nor were there any significant between-diet differences within the ISCH regions of IPC + ischaemia hearts. Concentrations of LPO and MDA in ISCH regions were significantly lower in IPC + ischaemia hearts than in ischaemia only hearts (*p* < 0.0001). Pairwise comparison indicated that this IPC difference was evident in SF and *n*-6 PUFA diets only.

Myocardial MnSOD activity was significantly greater in ISCH compared to non-I regions of hearts from SF and *n*-6 PUFA fed rats but not significantly changed within LC*n*-3 PUFA hearts (Figure 3C). The perfusion protocol incorporating IPC + ischaemia had no different effect on MnSOD activity to ischaemia alone.

### 3.4. Infarct

In hearts subjected to the ischaemia perfusion protocol, infarct size was significantly smaller in LC*n*-3 PUFA hearts (ischaemia infarct size (% Iz/r): SF 50 ± 1 *n* = 6; *n*-6 PUFA 47 ± 1 *n* = 6; LC*n*-3 PUFA *n* = 6 11 ± 1 *n* = 6, (*p* < 0.05)). In hearts subjected to the IPC + ischaemia protocol, the infarct size was significantly smaller in the SF and *n*-6 PUFA hearts than in the corresponding ischaemia group (*p* < 0.05). There was no significant difference within the LC*n*-3 PUFA diet. (IPC + ischaemia infarct size (% Iz/r): SF 13 ± 1 *n* = 6; *n*-6 PUFA 12 ± 1 *n* = 6; LC*n*-3 PUFA 10 ± 1 *n* = 6).

### 3.5. Associations between Oxidation Biomarkers, Antioxidant and Infarct Size

**Figure 4:**
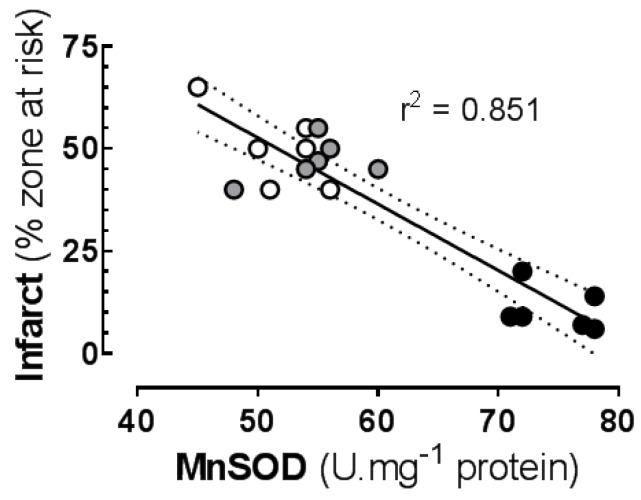
Correlation between: basal (non-I) concentration of superoxide dismutase; and infarct size in isolated rat hearts subjected to 30 min index ischaemia and 120 min reperfusion. Rats fed supplemented diets for six weeks—open symbols: saturated fat (SF) diet; shaded symbols: *n*-6 PUFA diet; closed symbols: LC*n*-3 PUFA fish oil diet.

*Ischaemia protocol*: Infarct size was positively associated with lipid oxidation biomarker production in the ISCH region, independent of diet (Table 2). The acute increases in LPO and MDA (ISCH compared to the non-I region) correlated better than the absolute ISCH concentrations of LPO and MDA. Lipid oxidation biomarkers LPO and MDA were correlated in the ISCH region. Ischaemic production of LPO and MDA and infarct size were inversely associated with the basal (non-I) MnSOD activity (Table 2). The strongest association was the inverse correlation between basal MnSOD and infarct size (Figure 4).

*IPC*: In hearts subjected to the IPC + ischaemia perfusion protocol, infarct size was not significantly correlated with LPO, MDA or MnSOD concentrations, and ischaemia-induced increase in MDA but not LPO was correlated with MnSOD activity. Lipid oxidation biomarkers LPO and MDA were correlated in the ISCH region (Table 2).

Pooled analysis of data from both perfusion protocols revealed significant correlations of infarct size with lipid oxidation markers and with MnSOD. Lipid oxidation biomarkers LPO and MDA were correlated in the ISCH region (Table 2).

## 4. Discussion

A diet rich in LC*n*-3 PUFA from fish oil modified the fatty acid profile of myocardial membrane phospholipids, increasing the percentage of fat as DHA and the peroxidisability index (predicting an increase in risk of oxidative damage), yet paradoxically reduced the measured oxidative damage following I/R. While the increased myocardial peroxidation potential was associated with an increase in basal fatty acid peroxidation, confirming effects of DHA feeding recorded in plasma and liver [24], it also induced a marked chronic increase in MnSOD (endogenous antioxidant) activity, and inhibited I/R-induced lipid oxidation and infarction. Reactive oxygen species act as both the agents of damage and of conservation in IPC, causing cellular damage yet triggering protective signalling processes [25]. In this respect, LC*n*-3 PUFA supplementation reflects both the low level generation of ROS through lipid peroxidation [26] and up-regulation of endogenous antioxidants that are implicated as triggers and mediators respectively of late phase IPC [27]. This aligns fish oil nutritional preconditioning [5] not only with this more persistent form of IPC (variously known as late, delayed or second window of IPC), but through LC*n*-3 PUFA continuous presence as a membrane component, it also provides a persistent tolerance to I/R injury. This persistent preconditioning is also observed with repeated stresses like exercise and heat exposure [12]. In contrast, early phase IPC did not acutely affect basal lipid oxidation or antioxidant activity during the 150 min post preconditioning time course of this study protocol. Moreover, IPC prevention of lipid oxidation and infarction during the index ischaemia was not additive to the effects of fish oil feeding. Admittedly the anti-infarct effects of both fish oil and IPC could be individually regarded as already maximal.

Fish oil-induced chronic increases in basal lipid oxidation directly correlated with basal MnSOD antioxidant activity in myocardium, which in turn was negatively correlated with the I/R-induced increase in lipid oxidation. This interdependence, which reflects the contrasting damaging influence and homeostatic signalling roles of ROS in ischaemia and IPC, can explain some of the lack of consistent correlation between oxidation products and anti-oxidants and sometime failure of oxidation markers to serve as clear criteria for defining oxidative stress [26]. The effects were consistent on LPO (an intermediate common to oxidation of all PUFA) and MDA (a stable end product of a single pathway also not specific for any PUFA family). Ultimately the infarct size was negatively correlated with MnSOD activity and directly correlated with the increase in lipid oxidation products in the ISCH region. Chronic elevation of plasma MnSOD has been previously observed during fish oil feeding [28], consistent with its persistent elevation over several days following multiple exposures to TNFα, exercise stress or heat stress [12].

Fish oil induced increases in antioxidant expression and reduced lipid peroxidation products were also reported in hepatic and renal tissue of immune suppressed mice [29,30] and hepatic tissue of hypertensive rats [31], conditions associated with heightened oxidative stress. In those studies, the fish oil diets were effective independently of varied provision of high or low concentrations of natural antioxidants in comparative *n*-6 PUFA or MUFA enriched diets. In the current study, the *n*-6 PUFA rich diet with its elevated vitamin E content did not change the membrane fatty acid composition sufficiently to modulate either membrane peroxidisability index or endogenous antioxidant enzyme activity relative to the low PUFA saturated fat enriched diet, and hearts from those diets were equally highly susceptible to oxidative damage. This is consistent with previous findings that both membrane effects and cardioprotective effects of *n*-6 PUFA are readily lost as the PUFA content is diluted by other fat sources [32,33]. This is not the case for LC*n*-3 PUFA, which sustain membrane composition [34,35], and cardiac [5,10,32,33,36] and other functional effects [35] to very low dietary concentrations. The ability of low (nutritionally relevant) intakes of fish oil to modify membrane composition and cardiac function, including prevention of I/R arrhythmias is important, since the provision of extremely high LC*n*-3 PUFA intakes can be pro-arrhythmic (fish oil concentrate 4 g/d/20 kg dog, equivalent to ≥40/d standard fish oil capsules in an 80 kg man) [37], perhaps representing the harmful effects of excessive oxidation. Similarly, in a senescence-prone mouse model, high fish oil feeding in conjunction with high total PUFA enhances oxidative stress and decreases lifespan [38].

The present study suggests that LC*n*-3 PUFA exert protection from ischaemia by activating signalling pathways that resemble those involved in late IPC or exercise, and we describe this as “nutritional preconditioning”. The current study used a LC*n*-3 PUFA intake equivalent to more than 30 g of fish oil per day in humans [34]. However, even very low doses in the range 0.16%–1.25% FO markedly increase myocardial DHA and peroxidation index (at 0.31% dietary fish oil equivalent to human 1–2 fish meals per week) DHA is increased from 7.7% to 14.9% of phospholipid fatty acids and PI is increased from 149 to 164 (calculated from Slee [34]). This is a dose that modulates skeletal muscle membrane fatty acids and muscle fatigue [35]. In skeletal muscle, reactive oxygen species capable of causing cellular damage when in physiological excess can at lower levels also act to optimise contractile performance and initiate long-term protective adaptations to the intermittent stress imposed by exercise training [39].

The present study deliberately used a high DHA fish oil, which does not reflect the composition of most nutritional supplement fish oils, but rather reflects the main LC*n*-3 PUFA derived from eating fish [4,40]. As the most abundant *n*-3 PUFA found in myocardium, DHA is also the main fatty acid underpinning the cardiac effects of fish and fish oil [4]. The use of two diets for comparison with fish oil allows specific attribution of the effects of fish oil feeding to its LC*n*-3 PUFA content, since similar total PUFA content was provided in the *n*-6 and LC*n*-3 PUFA diets; similar *n*-6 PUFA was provided in the SF and LC*n*-3 PUFA diets; and low saturated fat was provided in the *n*-6 PUFA diet, all without effect.

The increased expression of antioxidants within LC*n*-3 PUFA hearts was restricted to the mitochondrial form of SOD (MnSOD or SOD2) with CuZnSOD and GSx unchanged. This suggests localisation of the primarily influence of LC*n*-3 PUFA to the mitochondria. Increased MnSOD activity is similarly selectively implicated in the sustained cardioprotection elicited by heat stress and in late, delayed or second window of IPC [41], whereas over-expression of cardiac MnSOD in mice enhances contractile function, slows heart rate and increases efficiency of myocardial O_2_ consumption [42], all properties shared by dietary fish oil [5,43,44]. Furthermore, the fish oil-reduced cardiac oxygen consumption and reduced susceptibility to I/R-damage and arrhythmias in rats is linked to mitochondrial Ca^2+^ handling [44]. In contrast, early IPC inhibited acute lipid oxidation and infarction did not involve upregulation of mitochondrial SOD, confirming its difference from late IPC [41] and highlighting a difference to the more persistent forms of preconditioning including exercise [45,46,47], late IPC [41] and now fish oil-induced nutritional preconditioning.

This study confirmed that increasing myocardial membrane percentage content of long chain *n*-3 highly polyunsaturated fatty acids by feeding fish oil, increased the basal peroxidation of cellular fatty acids, which in turn increased the activity of endogenous mitochondrial antioxidant superoxide dismutase. When these hearts were acutely subjected to regional I/R, the stimulated lipid oxidation and myocardial damage were reduced. The increase in peroxidation index of myocardial membranes through fatty acid compositional change and associated chronic mild elevation in lipid peroxidation products provokes a persistent physiological stress that might better be described as “oxidative shielding” [48], which if confirmed at lower fish oil intakes, could explain much of the cardioprotective effect of regular fish consumption. This readily available and safe nutritional approach appears to represent a natural form of late preconditioning, which, characterised by its persistence over time, would be particularly valuable in the clinical setting, where oxidative insults occur unexpectedly and preclude the use of planned preventative interventions [41]. The observation, however, also raises the possibility that like exercise training [45], effects of fish oil nutritional preconditioning may be blunted by concomitant antioxidant supplementation.

## Figures and Tables

**Table 1:** Influence of dietary fat (six weeks) on phospholipid fatty acid composition as percentage of total phospholipid fatty acids of rat heart ventricle.

				DIET					
Fatty Acid	SF			*n*-6 PUFA			LC*n*-3 PUFA		
16:0	9.7	±	0.1	10.2	±	0.2	10.8	±	0.1
18:0	23.7	±	0.2	23.8	±	0.1	22.4	±	0.2
18:1*n*-9	^a^ 9.5	±	0.1	^b^ 5.4	±	0.1	^b^ 4.3	±	0.3
18:1*n*-7	3.6	±	0.1	3.5	±	0.1	3.4	±	0.1
Total SFA	33.80	±	0.13	34.70	±	0.80	33.70	±	0.40
Total MUFA	^a^ 13.50	±	0.12	^b^ 8.95	±	0.30	^b^ 7.75	±	1.10
18:2*n*-6 (LA)	^b^ 17.50	±	0.20	^a^ 18.7	±	0.40	^c^ 5.60	±	0.03
20:4*n*-6 (AA)	^a^ 23.30	±	0.30	^a^ 23.5	±	0.20	^b^ 13.30	±	0.15
22:5*n*-6	n.d			^a^ 1.50	±	0.12	^a^ 1.06	±	0.05
20:5*n*-3 (EPA)	n.d			n.d			1.30	±	0.01
22:5*n*-3 (DPA)	1.90	±	0.04	1.02	±	0.02	1.17	±	0.04
22:6*n*-3 (DHA)	^b^ 12.20	±	0.04	^b^ 10.02	±	0.20	^a^ 28.30	±	0.04
Total (*n*-6) PUFA	^b^ 40.80	±	0.20	^a^ 43.80	±	0.60	^c^ 20.00	±	0.16
Total (*n*-3) PUFA	^b^ 14.10	±	0.06	^c^ 11.00	±	0.20	^a^ 30.70	±	0.08
Total PUFA	54.90	±	4.50	54.70	±	4.50	50.70	±	4.40
UI	^b^ 215.40	±	1.20	^b^ 215.10	±	0.50	^a^ 260.58	±	1.20
Peroxidisability Index	^b^ 156.20	±	1.20	^b^ 149.50	±	1.60	^a^ 201.10	±	0.70

SF: saturated fat enriched diet; *n*-6 PUFA: *n*-6 PUFA enriched diet; LC*n*-3 PUFA: *n*-3 PUFA enriched diet; SFA: saturated fatty acids; MUFA: monounsaturated fatty acids; PUFA: polyunsaturated fatty acids; LA: linoleic acid; AA: arachidonic acid; EPA: eicosapentaenoic acid; DPA: docosahexaenoic acid; DHA: docosahexaenoic acid. Unsaturation index (UI) was calculated according to the formula: UI = 1 × (% monoenoic acids) + 2 × (% dienoics) + 3 × (% trienoics) + 4 × (% tetraenoics) + 5 × (% pentaenoics) + 6 × (% hexaenoics) or sum (fatty acid percent) × (number of double bonds). Peroxidatisability index was calculated from the formula: (% dienoic acids × 1) + (% trienoics × 2) + (% tetraenoics × 3) + (% pentaenoics × 4) + (% hexaenoics × 5) [17]. n.d: not detected. *n* = 6 per dietary group. ^a, b, c^ Values not sharing a common letter superscript are significantly different (ANOVA, *p* < 0.05).

**Table 2:** Correlations between lipid oxidation products, antioxidants and infarct size.

Dependent Variable	Independent Variable	Association	*r*^2^	*p* for Slope
**Ischaemia Protocol**				
Infarct	LPO (ISCH)	positive	0.337 *	0.018
Infarct	LPO increase	positive	0.478 **	0.004
Infarct	MDA (ISCH)	positive	0.356 *	0.015
Infarct	MDA increase	positive	0.517 **	0.004
Infarct	MnSOD (basal)	negative	0.851 **	<0.0001
MDA (ISCH)	LPO (ISCH)	positive	0.481 **	0.006
LPO increase	MnSOD (basal)	negative	0.397 **	0.009
MDA increase	MnSOD (basal)	negative	0.617 **	0.001
**IPC + Ischaemia Protocol**				
Infarct	LPO (ISCH)	positive	0.039	0.483 n.s.
Infarct	LPO increase	positive	0.147	0.175 n.s.
Infarct	MDA (ISCH)	positive	0.175	0.150 n.s.
Infarct	MDA increase	positive	0.009	0.728 n.s.
Infarct	MnSOD (basal)	negative	0.058	0.335 n.s.
MDA (ISCH)	LPO (ISCH)	positive	0.764 **	<0.0001
LPO increase	MnSOD (basal)	negative	0.128	0.174 n.s.
MDA increase	MnSOD (basal)	negative	0.293 *	0.017
**Overall**				
Infarct	LPO increase	positive	0.583 **	<0.0001
Infarct	MDA increase	positive	0.475 **	<0.0001
Infarct	MnSOD (basal)	negative	0.270 *	0.0012
MDA (ISCH)	LPO (ISCH)	positive	0.760 **	<0.0001

LPO: lipid hydroperoxides. MDA: malondialdehyde. MnSOD: manganese superoxide dismutase. ISCH: ischaemic region. Basal: non-ischaemic region of ventricle wall. n.s.: not significant (*p* > 0.05); * *p* < 0.05; ** *p* < 0.01.
